# Comparison of Fiberoptic-Guided Tracheal Intubation Through Intubating Laryngeal Mask Airway (ILMA) FastrachTM and Ambu® Aura-i™: A Randomized Clinical Study

**DOI:** 10.7759/cureus.10178

**Published:** 2020-09-01

**Authors:** Navya Mishra, Avnish Bharadwaj

**Affiliations:** 1 Anesthesiology, Mahatma Gandhi Medical College and Hospital, Jaipur, IND

**Keywords:** ambu aura-i®, ilma, intubation, supraglottic airway device, fibreoptic

## Abstract

Background and aim

The primary aim of the study was to compare the intubation characteristics and effectiveness of intubating laryngeal mask airway (ILMA) and Ambu® Aura-i™ as a conduit for facilitating fiberoptic-guided intubation.

Methods

Eighty patients were enrolled in the randomized-controlled hospital-based study. After inducing general anesthesia, an appropriately sized ILMA (group 1)/Ambu Aura-I (group 2) was placed. Fiberoptic assessment of the glottic view was done followed by fiberoptic-guided tracheal intubation. The time taken for the insertion of ILMA/Ambu Aura-i, glottic view grading, time taken for fiberoptic-guided intubation, ease of intubation, time taken for the removal of ILMA/Ambu Aura-i were recorded. Data analysis was done using the two-tailed independent t-test, paired t-test, and Fisher’s exact probability test.

Result

Anthropometric and airway parameters were similar in both groups. The success rate of the insertion of both devices was 100%. In group 1, the mean time taken for the insertion was 20.53±1.91, and it was 13.98±2.4 in group 2 (P<0.001S). Fiberoptic assessment of the glottic view in group 1 (ILMA group) was grade 1 in 80% of the patients, whereas it was 92% in group 2 (Ambu Aura-i) (P=0.54). The mean time taken for fiberoptic-guided intubation was 14.95±1.85 in group 1 and 14.15±1.37 in group 2 (P>0.001). No significant difference was observed according to the number of attempts in intubating through ILMA/Ambu Aura-i. Seventy-five percent (75%) in group 1 and 87.5% in group 2 were successfully inserted on the first attempt (𝑝 = 0.33). The time taken for the removal of the device was 11.87 +1.265 seconds in group 1 and 11.25±1.58 seconds in group 2 (P=0.054).

Conclusion

The Ambu Aura-i scores superiorly over ILMA in requiring less time for successful insertion on the basis of statistical analysis and hence appears to be a better independent ventilatory device.

## Introduction

Airway management is one of the most challenging tasks for anesthesia and critical care providers. Brain et al. (1997) bioengineered a new laryngeal mask prototype, intubating laryngeal mask airway (ILMA), which is an intubating system that eliminated the need for anatomical distortion and did not require the manipulation of the head and neck, thus increasing its utility in patients with cervical spine pathology [1].

Ambu® Aura-i™ is a new second-generation magnetic resonance compatible, phthalate-free, disposable supraglottic airway device designed to allow the passage of conventional cuffed tracheal tubes in patients of all age groups. It is available in various sizes based on the bodyweight of patients, ranging from <5 - >90 kg and is a suitable conduit for fiberoptic-guided intubation. The primary aim of the study was to compare the intubation characteristics and effectiveness of ILMA and Ambu Aura-i as a conduit for fiberoptic-guided intubation through it in terms of the glottic view on fiberoptic examination, time taken for fiberoptic bronchoscope (FOB)-guided intubation (seconds), ease of intubation, the success rate of intubation, and time taken for the removal of the device over the tracheal tube (seconds).

## Materials and methods

With the approval of the institute’s ethics committee (MGMCH Ethics Committee no. MGMCH/IEC/JPR/2017/323 dated March 3, 2017) and prior written informed consent, 80 consenting adult patients of either gender, weighing 50-70 kg, belonging to the American Society of Anesthesiologists (ASA) I-II, having mouth opening >2.5 cm, and undergoing elective major surgery requiring tracheal intubation were randomly selected. The study was conducted in the department of anesthesiology, Mahatma Gandhi Medical College & Hospital, Jaipur. All the participating patients underwent a thorough preoperative examination a day before surgery, with special attention paid to airway indices, including mouth opening, Mallampatti scoring, thyromental distance, sternomental distance, and interdental distance. We recorded demographics, including the age, weight, height, and gender of all the patients, and explained the details of the procedure to them before exercising the option to participate in the study.

Patients were excluded from the study if they had a cardio-respiratory disease (hypertension, chronic obstructive airway disease, ischemic heart disease), cerebrovascular disease, reflux esophagitis, hiatus hernia, peptic ulceration, or had not fasted. Patients with predicted difficult airway or inadequate mouth opening were also excluded.

On the day of surgery, on arrival to the operation room, monitoring was set up, including electrocardiogram (ECG), non-invasive blood pressure, end-tidal carbon dioxide (EtCO2) measurement, and pulse oximetry. An intravenous (IV) line was secured and baseline parameters were recorded. Patients were pre-medicated with Inj. midazolam 0.02 mg/kg, Inj. fentanyl 1 mcg/kg, Inj. glycopyrrolate 0.2 mg intravenously.

Before the induction of anesthesia, patients were randomized using the chit and box method for group assignment:

Group 1: Control group (n=40) - Intubating LMA was used for airway management

Group 2: Test group (n=40) - Ambu Aura-i was used for airway management

A standard technique for anesthesia was followed. Pre-oxygenation with 100% oxygen was done via a facemask for 3 min. Anesthesia was induced with intravenous propofol 2.5 mg/kg IV 60 seconds after IV bolus of Inj. Xylocard 1.0 mg/kg.

In group 1 patients, ILMA was inserted on the disappearance of the eyelash reflex when the jaw was completely relaxed. The standard technique of single-handed ILMA insertion was used as recommended by the manufacturers. In group 2, Ambu Aura-i was used for airway management. An additional bolus of propofol 10-20 mg was administered if required. Successful insertion of the ILMA/Ambu Aura-i was judged by the observation of normal capnogram (EtCO2)>30 mmHg with a rectangular curve). The time taken for the insertion of ILMA/Ambu Aura-i was measured from the time ILMA/Ambu Aura-i was picked up to the appearance of the capnographic waveform after the first manual breath following ILMA/Ambu Aura-i insertion. Failure of ventilation through the ILMA/Ambu Aura-i produced resistance, leakage, and/or an abnormal capnograph (non-rectangular capnograph with (EtCO2) <30 mmHg), extension or flexion maneuver, or Chandy’s maneuver were carried out in that sequence.

A maximum of three attempts was allowed for mask insertion. In between the attempts, patients were oxygenated with a face mask. After three attempts or desaturation <90%, patients were moved to a supine position and intubated via conventional laryngoscopy. Such cases were classified as the failure of mask insertion. Failure to achieve a normal capnograph following spontaneous or controlled breath were also treated as the improper placement of the intubating laryngeal mask/Ambu Aura-i and tracheal intubation was performed with direct laryngoscopy in the supine position.

On achieving satisfactory ILMA/Ambu Aura-i placement, Inj. Atracurium 0.6 mg/kg IV was administered and intermittent positive-pressure ventilation (IPPV) with nitrous oxide + oxygen and minimum alveolar concentration (MAC) doses of isoflurane done.

After three minutes, ventilation through the ILMA/Ambu Aura-i was interrupted to allow the assessment of the glottic view and fiberoptic-guided intubation. The fiberoptic assessment of the glottis view was done by an independent investigator using a Storz flexible intubating fiberscope (Karl Storz SE & Co. KG, Tuttlingen, Germany) preloaded with well-lubricated special cuffed flexometallic tracheal tube of 7.0/7.5 mm size with its connector removed; the main investigator remaining blinded to the view of the glottis.

The glottis view was assessed and graded as follows:

GRADE 1 = only larynx seen

GRADE 2 = larynx and epiglottis posterior surface seen

GRADE 3 = larynx and epiglottis tip of the anterior surface seen, <50% visual obstruction of the epiglottis to the larynx

GRADE 4 = epiglottis downfolded and its anterior surface seen, >50% visual obstruction of the epiglottis to the larynx

GRADE 5 = epiglottis downfolded and larynx cannot be seen.

If needed, any maneuver needed to improve the glottis view under fiberoptic vision was performed and noted by the independent investigator.

On achieving optimum glottis view, fiberoptic-guided tracheal intubation was done with the preloaded tracheal tube as per the recommendation of the manufacturer. The tracheal tube connector was reconnected, and further ventilation with N_2_O+oxygen and isoflurane was done.

If intubation via the ILMA/Ambu Aura-i failed, tracheal intubation with direct laryngoscopy was performed in the supine position.

The time taken in intubation was defined as the time from insertion of the tip of the FOB into the metallic end of the intubating laryngeal mask to the appearance of the capnographic waveform after the first manual ventilation via the flexometallic tube following its final placement through the ILMA/Ambu Aura-i after the reconnection of the tracheal tube connector. After the successful placement of the tracheal tube, the intubating laryngeal mask was removed using the stabilizing rod for railroading the ILMA (a plain tracheal tube one size smaller was used in the case of Ambu Aura-i), holding the tracheal tube by the other hand to prevent accidental extubation. Proper tracheal placement of the tracheal tube was confirmed by capnography and tracheal tube secured with adhesive tape.

Time taken in the removal of the device was recorded as time from the disconnection of ventilation till recommencing ventilation after successful ILMA/Ambu Aura-i removal. This was the endpoint of the study.

Further ventilation was continued with maintenance anesthetics (N_2_O + Oxygen + Isoflurane + Atracurium). Anesthetics were stopped at the end of the surgery. The reversal of the neuromuscular block was done and extubation was done in the standard manner.

The following data were recorded for comparison between the groups: age, sex, weight, height, and known airway difficulty, Mallampati grades, and thyromental distances. The difficulty experienced in mask insertion, the number of attempts, number/type of adjusting maneuvres, and time needed for inserting the ILMA and placement of the tracheal tube (via the intubating laryngeal mask/ Ambu Aura-i), together with complications arising from these maneuvres. The Cormack and Lehane laryngoscopy score was noted for those patients who required intubation with direct laryngoscopy. Hemodynamic parameters, viz. heart rate, blood pressure, oxygen saturation (SpO2), and EtCO2, recorded at different time intervals.

**Figure 1:**
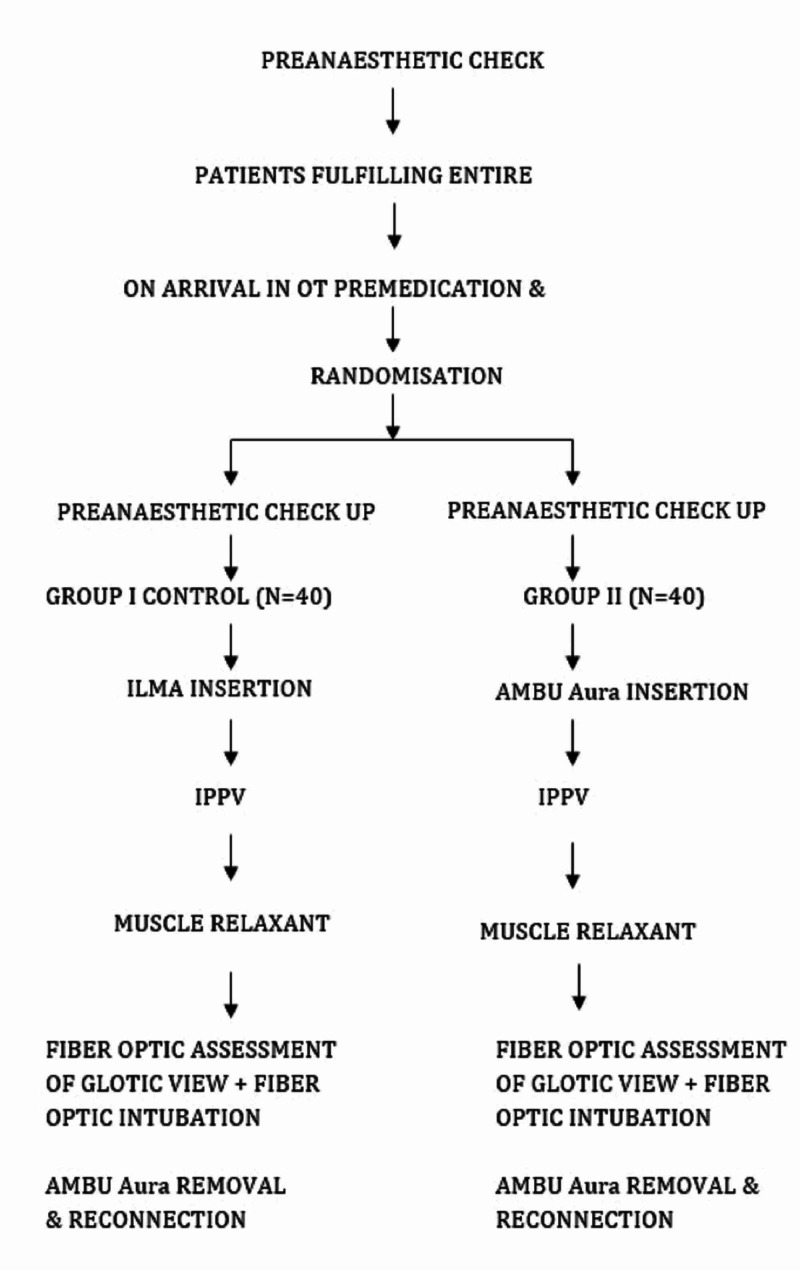
Flow chart describing the study

All the observations were recorded in a specially prepared proforma and a flow chart describing the study is provided (Figure 1).

Statistical analysis of the descriptive data was done.

## Results

All anthropometric parameters were similar in both groups. We were able to successfully insert the ILMA/Ambu Aura-i in 100% of the patients, i.e., in 80/80 patients. Out of these, in 83% of patients, the intubation was done at the first attempt, in 12% at the second attempt, and in 5% at the third attempt.

In our study, out of 40 patients intubated through ILMA (group 1), 37 had easy placement of ILMA, and we encountered some difficulty in mask placement in three cases. whereas out of the 40 patients who were intubated using Ambu Aura-i (group 2), 38 had easy placement of Ambu Aura-i, and only two had difficult insertion of Ambu Aura-i. Overall, the difference was insignificant.

The mean time taken for the insertion of ILMA was 20.53±1.91 sec for ILMA and 13.98±2.4 sec for Ambu Aura-i, respectively. More time was required in group 1 for successful ILMA insertion as compared to the time taken for Ambu Aura-i insertion in group 2 (P<0.001), which is statistically significant.

In our study, in group 1 (ILMA group), the glottis view was reported as grade 1 in 80% of the patients, whereas in group 2 (Ambu Aura-i), the glottis view was reported as grade 1 in 92% of the patients. The difference was not significant.

No significant difference was observed according to the number of attempts in intubating through ILMA/Ambu Aura-i among the groups. Seventy-five percent (75%) in group 1 and 87.5% in group 2 were successfully inserted at the first attempt itself.

(𝑝 = 0.33)

The mean time taken for fiberoptic-guided intubation was 14.95±1.85 seconds in group 1 (ILMA group) and 14.15±1.37 in group 2 (Ambu Aura-i® group). The difference was statistically insignificant.

The time taken for the removal of ILMA/Ambu Aura-i was similar in the two groups: 11.87 + 1.265 seconds for ILMA as compared to 11.25±1.58 seconds for Ambu Aura-i.

The extension maneuver was recruited in four out of 40 cases in each group, and no significant difference was observed on this count.

The mean heart rate in both groups exhibited a significant increase after premedication as compared to before premedication baseline values (p<0.001); the trend persisted until the accomplishment of mask insertion. Thereafter, the heart rate declined.

The insertion of ILMA or Ambu Aura-i and intubation through them per se did not result in a statistical change in heart rate from preceding values. Changes in heart rate at different time intervals were statistically similar in both groups.

Propofol injection decreased the systolic, diastolic, and mean blood pressure in both groups.

ILMA/Ambu Aura-i insertion or fiberoptic-guided intubation did not result in any statistically significant response on blood pressure as compared to baseline values.

The mean values of systolic, diastolic, and mean blood pressure are similar in the two groups at different time intervals. No significant difference in EtCO2 and SpO2 was observed between group 1 and group 2.

No incidence of esophageal intubation was encountered in any patient in our study and no instance of accidental extubation was recorded in any patient in group 1 as well as group 2.

No incidence of SpO2 desaturation, laryngospasm, aspiration, or any major adverse event has been recorded by us in both study groups.

See Tables 1-8 for more details.

**Table 1:** Anthropometric measurements BMI: body mass index

Age	Group 1	Group 2	
	36.33	36.15	
MEAN			
Gender (%age)	52.5	42.5	
Female			
Male	47.5	57.5	
Height	168.68	167.35	
MEAN			
Weight	64.48	64.48	
MEAN			
BMI	22.75	23.08	
MEAN			

**Table 2:** Ease of insertion of ILMA/Ambu Aura-i ILMA: intubating laryngeal mask airway

Ease of insertion	Group 1	Group 2
Easy	37	38
Difficulty	3	2

**Table 3:** Time taken in seconds for the insertion of ILMA/Ambu Aura-i ILMA: intubating laryngeal mask airway

Time Taken in Seconds	Group 1	Group 2	P-Value
Mean±SD	20.53±1.91	13.98±2.41	<0.001

**Table 4:** Distribution of the cases according to No of attempts of insertion of ILMA/Ambu Aura-i ILMA: intubating laryngeal mask airway

No. of Attempts	Group 1		Group 2	
	No.	%	No.	%
1	33	82.5	35	87.5
2	5	12.5	4	10
3	2	5	1	2.5
Total	40	100	40	100

**Table 5:** Table showing fiberoptic assessment of grading of the glottic view in group I and group 2

Fiberoptic Intubation	Group 1		Group 2		P-Value
Glottic View Grade	No	%	No	%
Grade1	32	80	35	87.5	0.54
Grade2	8	20	5	12.5	
Grade3	0	0	0	0	
Grade4	0	0	0	0	
Grade 5	0	0	0	0	

**Table 6:** Number of attempts for fiberoptic-guided intubation

Number of Attempts	Group 1		Group 2		P-Value
	No.	%	No.	%	0.332
1^st^	30	75	35	87.5	
2^nd^	7	17.5	4	10	
3^rd^	3	7.5	1	2.5	

**Table 7:** Time taken for fiberoptic-guided intubation

Time Taken	Group 1		Group 2		P-Value
	Mean	SD	Mean	SD	
Seconds	14.95	1.85	14.15	1.37	0.03

**Table 8:** Distribution of the cases according to the time taken for the removal of ILMA/Ambu Aura-i ILMA: intubating laryngeal mask airway

Time taken for the removal of ILMA	Group 1		Group 2		P-Value
	Mean	SD	Mean	SD	
Seconds	11.87	1.265	11.25	1.58	0.054

## Discussion

Intubating LMA has been found to be an effective ventilator device and tool for blind, as well as guided, tracheal intubation in several hospital-based studies with a high success rate. Ambu Aura-i is an excellent alternative to a face mask for achieving and maintaining control of the airway during routine and emergency anesthetic procedures in patients evaluated as eligible for a supraglottic airway or in situations where other attempts to establish an airway have failed. In view of the fact that there is a paucity of literature on the clinical use of Ambu Aura-i performance and clinical use in adults and only a few isolated case reports, along with controlled and comparative studies, with other supraglottic airway devices are available, we decided to add to the initial experience and conducted this hospital-based descriptive type of observational study.

The success rate of the insertion of a supraglottic airway device and intubation through it in our study was 100%. A maximum of three attempts was allowed and no significant difference was observed in the number of attempts taken for the insertion of ILMA/Ambu Aura-i. No significant difference was observed in the ease of insertion of the two devices and an extension maneuver was used only in four out of forty patients in each group. Previous studies have found a success rate of 100% for the insertion of ILMA and 86% for the insertion of Ambu Aura-i [2]. Similarly, in a previous study, a 100% success rate was reported in fiberoptic-guided intubation through ILMA [3]. In a previous study, it was found that the overall success rate of the insertion of the device after three attempts was more with Air-Q (Mercury Medical, Clearwater, FL) (96.6%) as compared with ILMA (91.6%) but overall, it was insignificant [4]. A previous study showed that ILMA placement was easy in 88.8% of cases, slightly difficult in 9.2%, while moderately difficult in 2% of cases but impossible in none of the cases​​​​​​ [5].​ Adjusting maneuvers used in the previous study were extension, flexion, and Chandy’s maneuver for adjusting ILMA insertion for successful ILMA placement [6].

The mean time taken in the insertion of ILMA was significantly higher than that observed in group 2. Similar observations were observed in a previous case study in which Ambu Aura Gain LM and LMA FastrachTM were compared [7]. A previous case study comparing Ambu Aura-i and LMA Supreme showed no significant difference in the time of insertion of both the device [8]. In our study, the glottic view was reported as grade 1 in 80% of patients in group 1 and 92% of patients in group 2. Similarly, in a previous case study using Ambu Aura-i, the glottic view was reported as grade 1 in 88% of patients and grade 2 in 10% of patients, whereas in 2% of patients, grade 3 was observed [2].^ ^Jagannathan et al. in their study also used similar criteria for grading the glottic view [9].

No significant difference was observed in the number of attempts taken for intubation through ILMA/Ambu Aura-i. Similar observations were reported in a previous study where ILMA and Ambu Aura-i was compared [8,10-11].

The mean time taken for fiberoptic-guided intubation in both groups showed no significant difference. Previous studies also reported a similar result [2,5,12]. No significant difference was observed in the time taken for the removal of ILMA/Ambu Aura-i. In our study, no incidence of complications such as esophageal intubation, accidental extubation, laryngospasm, or aspiration was reported. A previous study reported one case of accidental esophageal intubation while using Aura-i as a conduit for tracheal intubation ​​​​​​[13]​.^ ^One previous study reported accidental extubation during their study [6]. No significant difference was reported in hemodynamic parameters in both the groups. Similar observations were reported in the previous study [5].

The lower cost of Ambu Aura, its availability in sizes suitable for all age groups, supply in sterile and meant for single use only environment, magnetic resonance compatibility, and availability of a phthalate-free version may make it a more desirable and versatile ventilatory device and intubation tool for routine, as well as, emergency airway management.

## Conclusions

On the basis of the results obtained in our study, we infer that both ILMA as well as Ambu Aura-i are reliable and suitable conduits for fiberoptic-guided intubation through them. The intubation times with both are fairly speedy and clinically acceptable. Ambu Aura-i scores superiorly over ILMA in requiring less time for successful insertion on the basis of statistical analysis and hence appears to be a better independent ventilatory device.

## References

[1] Brain AIJ (1983). The laryngeal mask-a new concept in airway management. Br J Anaesth.

[2] Yahaya Z, Teoh WH, Dintan NA, Agrawal RA (2016). The Ambu Aura-i® laryngeal mask and LMA SupremeTM: a randomized trial of clinical performance and fibreoptic positioning in unparalysed, anaesthetised patients by novices. Anesthesiol Res Pract.

[3] Jagannathan N, Sohn LE, Sawardekar A (2012). A randomized trial comparing the Ambu ® Aura-i® with the air-QTm intubating laryngeal airway as conduits for tracheal intubation in children. Paediatr Anaesth.

[4] Malhotra S.K., Bharat KV, Saini V (2016). Comparison of success rate of intubation through Air‑Q. Indian J Anaesth.

[5] Baskett PJF, Parr MJA, Nolan JP (1998). The intubating laryngeal mask. Results of a multicentre trial with experience of 500 cases. Anaesthesia.

[6] Panwar M, Bhardwaj A, Chauhan G, Kalita D (2013). Intubating laryngeal mask airway as an independent ventilatory and intubating device. A comparison between supine, right lateral and left lateral position. Korean J Anaesthesiol.

[7] Preece G, Ng I, Lee K (2011). A randomised controlled trial comparing fibreoptic-guided tracheal intubation through two supraglottic devices: Ambu® Aura GainTM laryngeal mask and LMA® Fastrach™. Anaesth Intens Care.

[8] Beri N, Haleem S, Varshney VK, Fatima N (2017). Second generation extraglottic airway devices (Ambu, Air-Q,$ILMA): a comparative study to assess the relative success rate in first attempt and safety for blind tracheal intubation. Central Journal of ISA.

[9] Jagannathan N, Hajduk J, Sohn L (2015). A randomised comparison of the Ambu AuraGainTM and the LMA supreme in infants and children. Anaethesia.

[10] Jagannathan N, Sohn LE, Sawardekar A (2012). A randomized trial comparing the Ambu® Aura-i® with the air-QTm intubating laryngeal airway as conduits for tracheal intubation in children. Paediatr Anaesth.

[11] Joshi S, Sciacca RR, Solanki DR, Young WL, Mathur MM (1998). A prospective evaluation of clinical tests for placement of laryngeal mask airways. Anaesthesiology.

[12] Pandit JJ, Mac Lachlan K, Dravid RM, Popat MT (2002). Comparison of times to achieve tracheal intubation with three techniques using the laryngeal or Intubation laryngeal mask airway. Anaesthesia.

[13] de Lloyd LJ, Subash F, Wilkes AR, Hodzovic I (2015). A comparison of fibreoptic‐guided tracheal intubation through the Ambu®Aura‐i™, the intubating laryngeal mask airway and the i‐gel™: a manikin study. Anaesthesia.

